# Disconnected neuromagnetic networks in children born very preterm: Disconnected MEG networks in preterm children

**DOI:** 10.1016/j.nicl.2015.08.016

**Published:** 2015-09-01

**Authors:** Annette X. Ye, Michelle AuCoin-Power, Margot J. Taylor, Sam M. Doesburg

**Affiliations:** a Diagnostic Imaging, Hospital for Sick Children, Toronto, Ontario, Canada; b Institute of Medical Science, University of Toronto, Toronto, Ontario, Canada; c Neurosciences and Mental Health, Hospital for Sick Children Research Institute, Toronto, Ontario, Canada; d Department of Psychology, University of Toronto, Toronto, Ontario, Canada; e Department of Medical Imaging, University of Toronto, Toronto, Ontario, Canada; f Department of Paediatrics, University of Toronto, Toronto, Ontario, Canada; g Department of Biomedical Physiology and Kinesiology, Simon Fraser University, Vancouver, British Columbia, Canada; h Behavioural and Cognitive Neuroscience Institute, Simon Fraser University, Vancouver, British Columbia, Canada

**Keywords:** Magnetoencephalography, Resting state, Neural oscillations, Functional connectivity, Phase synchrony, Very preterm

## Abstract

Many children born very preterm (≤32 weeks) experience significant cognitive difficulties, but the biological basis of such problems has not yet been determined. Functional MRI studies have implicated altered functional connectivity; however, little is known regarding the spatiotemporal organization of brain networks in this population. We provide the first examination of resting-state neuromagnetic connectivity mapped in brain space in school age children born very preterm. Thirty-four subjects (age range 7–12 years old), consisting of 17 very preterm-born children and 17 full-term born children were included. Very preterm-born children exhibited global decreases in inter-regional synchrony in all analysed frequency ranges, from theta (4–7 Hz) to high gamma (80–150 Hz; *p* < 0.01, corrected). These reductions were expressed in spatially and frequency specific brain networks (*p* < 0.0005, corrected). Our results demonstrate that mapping connectivity with high spatiotemporal resolution offers new insights into altered organization of neurophysiological networks which may contribute to the cognitive difficulties in this vulnerable population.

## Highlights

• We recorded resting-state magnetoencephalography in school-age children born very preterm and healthy children.
• We examine functional connectivity across a wide frequency spectrum in brain space.
• Global reductions in neural synchrony were detected in children born very preterm.
• These reductions encompass networks related to executive function and overall cognitive flexibility.
• These effects may be relevant to cognitive and behavioural difficulties reported in the ex-preterm population.

## 1 Introduction

Very preterm birth has profound consequences for public health worldwide (Saigal and Doyle, 2008). Infants born very preterm (≤32 weeks gestation) now represent up to 2% of all live births, with rates steadily increasing. Despite ongoing improvements in the management of preterm infants, morbidity among these survivors remains high (Horbar et al., 2002). Approximately 50% of very preterm-born children exhibit neurodevelopmental impairments at school age, and up to two-thirds will need educational or psychological support during their school years (Larroque, 2011). Even when intelligence is broadly normal, selective difficulties in areas such as executive functions and visual perceptual abilities often become apparent at school age (Aarnoudse-Moens et al., 2009a,b, 2012; Anderson, 2014).

Advances in magnetic resonance imaging (MRI) have enabled a large body of work characterizing the effects of prematurity on brain structure and its relation to outcome (Ment et al., 2009). It is widely hypothesized that infants born very prematurely express atypical development of the subplate, a large transient cerebral structure maximal in the last trimester of gestation (Kostovic and Rakic, 1980). During this period, there is rapid growth of thalamocortical fibres and cortical dendritric trees, leading to a substantial increase in total cerebral volume from 28 to 40 weeks (Kapellou, 2006). At term-equivalent age, infants born preterm exhibit significantly reduced cortical grey matter volume (Soria-Pastor, 2009) and continue to display impaired cortical growth even in childhood and adolescence (de Kieviet et al., 2012) in the absence of significant medical complications. In volumetric MRI studies conducted at school age, very preterm-born children have reduced volumes of the basal ganglia (Peterson, 2000), amygdalae (Peterson, 2000), thalami (Lax, 2013), and hippocampi (Omizzolo, 2013). White matter development has also been shown to be altered in middle childhood (Duerden et al., 2013), indicating that structural connections among brain regions do not develop typically in this population (Miller and Ferriero, 2009). This has been attributed to heightened vulnerability of oligodendrocyte progenitor cells due to early exposure to the extrauterine environment.

To study the effects of prematurity on brain function, resting state functional MRI (fMRI) has been used to characterize BOLD signal correlations and their organization into large-scale resting state networks (RSNs). Altered functional connectivity of RSNs in children born very preterm is present in the neonatal period (Damaraju, 2010) and continues into adulthood (White, 2014). Although fMRI provides excellent spatial resolution, it has limited temporal resolution (<0.1 Hz), therefore preventing the measurement of neurophysiological oscillations occurring at faster time scales. This presents a critical gap in knowledge, as cognition and perception (Palva and Palva, 2007; Uhlhaas, 2009) are mediated by synchronous neuronal oscillations occurring across a broad frequency beyond that obtained using fMRI. Magnetoencephalography (MEG) offers a uniquely good combination of spatial and temporal resolution, thereby enabling the imaging of network interactions on a millisecond basis that are accurately resolved at the level of functional neuroanatomy.

MEG investigations have revealed atypical cortical responses in extremely preterm infants (Rahkonen, 2013) and altered task-dependent functional connectivity at school age (Doesburg, 2011a). Disruptions in resting state MEG oscillations have also been reported in very preterm children at school age (Doesburg, 2011b) and are associated with adverse neonatal experience (Doesburg, 2013). As previous investigations of atypical resting state MEG phase synchronization in very preterm children have been conducted exclusively at the sensor level, the relevance of specific brain regions and networks to altered connectivity remains poorly understood. The present study investigated resting state network synchrony among MEG signals reconstructed from brain regions throughout the cortex and sub-cortex. Middle childhood (7–12 years of age) represents a vital period of rapid neural development and coincides with the period when cognitive delays associated with preterm birth are most frequently identified (Fair, 2009; Giedd and Rapoport, 2010; Shaw, 2008). Based on prior studies in this population, we hypothesized that functional connectivity would be reduced in very preterm children in comparison to their full-term born peers at school age.

We describe, for the first time, global reductions in resting MEG synchrony in multiple frequency bands in very preterm-born children. We also show these reductions in connectivity manifest in different networks at particular frequencies, and suggest poor integration of neural networks are related to higher-order cognitive flexibility such as task/executive control, working memory, and visuospatial abilities. These findings of reduced neurophysiological network connectivity open new possibilities of linking adverse neonatal events with long-term cognitive and behavioural outcomes in this population.

## 2 Material and methods

### 2.1 Participants

Inclusion criteria for this study were age between 7 and 12 years at the time of testing and gestational age at birth ≤32 weeks for very preterm and ≥37 weeks for full-term born children. Exclusion criteria were a history of focal traumatic brain injury, cerebral palsy or other neurological diagnosis, motor or sensory impairments, the use of psychoactive medication, or a history or existing diagnosis of psychiatric disorder or learning disability. None of the children were diagnosed with autism spectrum disorder. The initial dataset contained 50 children (23 very preterm and 27 full-term born children). After age- and sex-matching, as well as exclusion for head motion in the MEG scanner, 34 subjects (17 very preterm and 17 term born children) were entered into the final analysis.

Participant demographic and behavioural data are shown in Table 1. Data regarding gestational age and birth weight was collected from all very preterm participants. None of the very preterm participants had history of cerebral palsy, grade III/IV intraventricular haemorrhage, or periventricular leukomalacia by retrospective chart review and parental questionnaire. All studies were performed with written informed parental consent and child assent and approval by the Hospital for Sick Children Research Ethics Board and the Declaration of Helsinki.

### 2.2 Data acquisition

MEG data were acquired using a third-order synthetic gradiometer configuration of 151-channel whole-head, adult-sized CTF system (CTF Systems Inc., Coquitlam, Canada). Subjects were supine in the scanner and viewed a centrally presented fixation cross while 5 minutes of data were recorded at a sampling rate of 600 Hz. Sponges were placed on both sides of the head to reduce head movement. Subjects were monitored via video and audio recording to ensure wakefulness and attentiveness. Head position was recorded continuously by measuring the location of three fiducial coils, located at the nasion and left and right preauricular points. Fiducial head coils were energized at 1470 Hz, 1530 Hz, and 1590 Hz, respectively. Immediately following the MEG recording, a 3 T structural MR image (MPRAGE) was acquired. MRI scans were read as normal for all participants by experienced paediatric neuroradiologists.

### 2.3 Behavioural data analyses

#### 2.3.1 Neuropsychological assessment

Participants were assessed using a battery of neuropsychological assessments consisting of a selection of subtests from the following: Wechsler Abbreviated Scale of Intelligence (WASI) (Wechsler, 2002), Working Memory Test Battery for Children (WMTB-C) (Pickering and Gathercole, 2001), NEPSY — Second Edition (NEPSY-II) (Korkman et al., 2007), and Behavior Rating Inventory of Executive Function (BRIEF) (Gioia and Isquith, 2000). We report scores from 12 subtests that characterize intelligence, working memory, executive functioning and social perception of the two groups (Table 2). To aid in interpretation, the typical data distributions associated with each test are as follows: WASI and WMTB-C (standard scores: mean = 100, standard deviation (SD) = 5); NEPSY-II (scaled scores: mean = 10, SD = 3); and BRIEF (T scores: mean = 50, SD = 10). A more detailed description of each subtest is provided in Supplemental Materials.

#### 2.3.2 Statistical analysis of neuropsychological data

Differences in neuropsychological variables between the very preterm and full-term groups were compared via unpaired two-tailed t-tests. All results were FDR-corrected for 12 tests (p < 0.05, corrected, q = 0.05) (Benjamini and Hochberg, 1995). Cohen's delta coefficient *d* served as a measure of effect size, with r = 0.20 representing small, r = 0.50 a medium, and r = 0.80 a large effect.

#### 2.3.3 Brain–behaviour relations

We assessed correlations between global functional connectivity and neuropsychological scores for the very preterm-born children using mean-centring partial least square (PLS) analysis. PLS analysis is a multivariate statistical technique that can be used to relate two sets of variables to each other, and is valuable when the sample sizes are not large (McIntosh and Lobaugh, 2004). Nonparametric resampling was used to assess statistical significance and reliability of experimental effects. We refer the reader to the original description of the technique for further details (McIntosh and Lobaugh, 2004; Lobaugh et al., 2001; McIntosh et al., 1996; McIntosh and Mišić, 2013).

### 2.4 MEG data analyses

#### 2.4.1 Data preprocessing

MEG data were preprocessed to verify data quality and to reduce contamination from artefacts. A third-order spatial gradient was applied to correct for environmental noise using the manufacturer's compensation system (CTF Systems Inc., Coquitlam, Canada). Recordings were band-pass filtered from 1 to 150 Hz with a notch filter at 60 Hz (8 Hz bandwidth). Participants with head movements greater than 10 mm for more than 10% of the recording were excluded from further analysis (n = 7), resulting in a total of 34 participants in the final study. This standard of tolerance is typical for MEG studies of paediatric populations, allowing collection of MEG data from a clinical population without creating a biased sample (Taylor et al., 2011).

#### 2.4.2 Source reconstruction

We reconstructed time series representing activity of multiple locations in the brain using a scalar beamformer (Cheyne et al., 2006). Beamformer analysis implements an adaptive spatial filter, where the aim is to estimate the signal from a given brain location through the weighted sum of surface field measurements while attenuating activity from other sources. Each participant's MEG data were co-registered with his/her individual MRI for accurate neuroanatomical localization. We used statistical parametric mapping (SPM2, Wellcome Department of Imaging Neuroscience, London, UK) for MR image preprocessing. Individual anatomical MR images were normalized into standard Montreal Neurological Institute space using a nonlinear transform in SPM2. Seed regions representing all 90 cortical and subcortical brain areas from the Automated Anatomical Labeling (AAL) atlas (Tzourio-Mazoyer, 2002) were then warped back into each individual's brain space (Table 3).

#### 2.4.3 Inter-regional phase synchrony

Data were filtered into theta (4–7 Hz), alpha (8–14 Hz), beta (15–30 Hz), low gamma (30–80 Hz), and high gamma (80–150 Hz) frequency ranges. For each frequency bin, phase synchrony between sources was estimated by computing a weighted phase lag index (wPLI) (Vinck et al., 2011) across all possible source pairs for the entire 5 minute recording, resulting in five 90 × 90 symmetric matrices for each subject. WPLI is a metric of phase synchrony that estimates non-zero phase lag interdependencies by weighting the contribution of the observed phase leads and lags by the magnitude of the imaginary component of the cross-spectrum between each pair of sources. WPLI values range between 0 and 1, with 0 indicating random distribution of phase and 1 indicating constant non-zero lag phase difference between sources. To ensure that the observed phase synchrony values were not attributable to systemic differences in distance of head to sensor array, we calculated the absolute value of the analytic signal across all sources for all five frequencies. No significant differences in global oscillatory power were found between very preterm-born and full-term born children (*p* > 0.05, Supplemental Materials).

#### 2.4.4 Global connectivity analysis

To test for overall group differences in connectivity within each analysed frequency range, we averaged across all source pairs for each matrix to obtain a single value representing global functional connectivity for each participant. Group differences at each frequency were evaluated using Mann–Whitney U tests. Bonferroni correction was applied to account for multiple comparisons across the frequency bins studied (i.e. threshold for significance, *p* < (0.05/N), where N = 5, thus *p* < 0.01).

#### 2.4.5 Group network differences

To compare connectivity at the network level between the very preterm and full-term groups, we used the Network Based Statistic (NBS) (Zalesky et al., 2010). In this context, a network refers to a contiguous set of inter-regional connections that differs between the very preterm and full-term groups. The NBS method protects against false positives due to multiple comparisons in brain network connectivity analysis. Since statistical significance is assigned at the level of the network as a whole, rather than at the level of each pairwise connection, the choice of the primary test statistic threshold only affects the sensitivity of the method. To target strong, topologically focal differences between groups, we chose conservative thresholds that were adapted to the data distribution under investigation (Zalesky et al., 2010, 2012). Specifically, significant group differences in network connectivity were evaluated using a primary threshold of *p* < 0.00004 in two-tailed t-tests, and then family-wise error (FWE) corrected at *p* < 0.0005 using 10,000 permutations. In the NBS method, effective control for multiple comparisons is achieved irrespective of this initial threshold selection (Zalesky et al., 2010, 2012). To ensure that the choice of statistical significance did not bias our findings, validation tests for the NBS analysis were performed with more liberal and typical FWE corrections at alpha levels of 0.05 and 0.001 (Supplemental Materials).

## 3 Results

### 3.1 Demographic and neuropsychological data

Table 1 describes the clinical and demographic characteristics for the very preterm and full-term groups. The two groups had similar proportions of males, and did not differ by age at scan. The very preterm-born group performed more poorly than full-term born participants on all measures of general intelligence and executive function (Table 2). Parents reported that children born very preterm were more likely to exhibit clinically significant behavioural problems as a result of executive dysfunction on the Metacognition Index (MI), Behavioural Regulation Index (BRI), and Global Executive Composite (GEC). None of the neuropsychological variables investigated were significantly different between very preterm-born and full-term participants at school age after correction for multiple comparisons (*p* > 0.05, FDR corrected).

### 3.2 Global and network-level connectivity differences

**Fig. 1:**
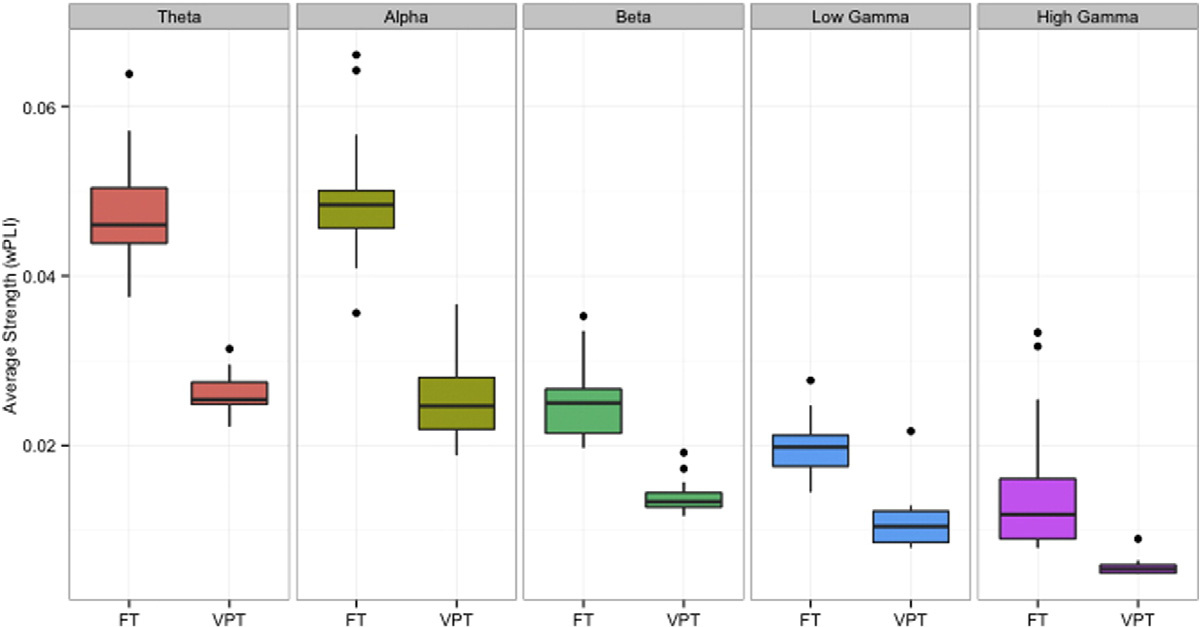
Average functional connectivity for each group and frequency range, measured by whole-brain average of synchrony between each pair of regions in the brain. Values represent the weighted phase lag index for theta (red), alpha (yellow), beta (green), low gamma (blue) and high gamma (purple) broadband frequencies. FT = full-term control subjects, VPT = very preterm-born subjects. Significant differences (*p* < 0.01, corrected) between groups are present at each frequency.

**Fig. 2:**
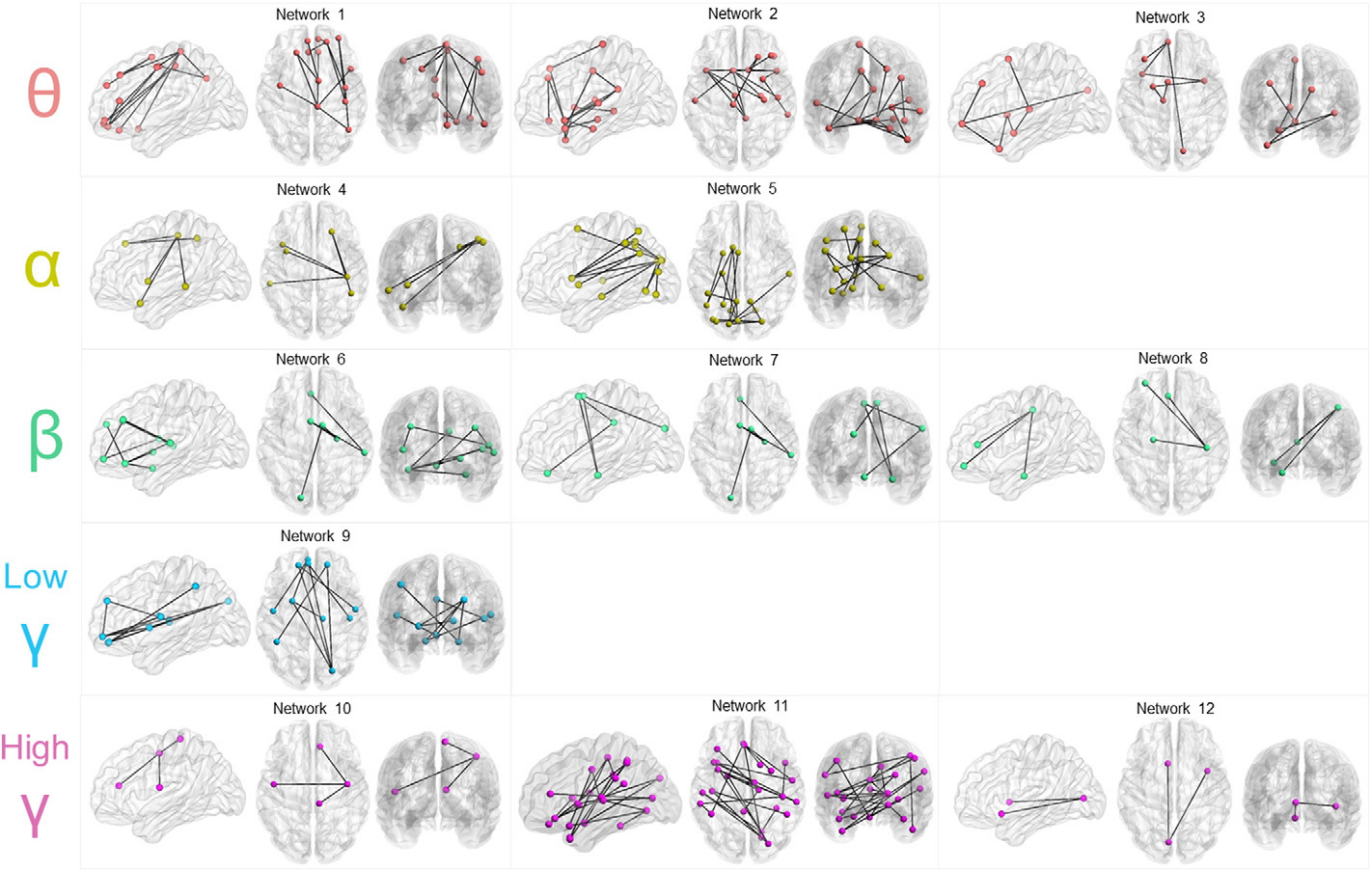
Very preterm-born (VPT) children demonstrated reduced network connectivity compared to full-term control children at various frequencies in 12 different networks (*p* < 0.0005, corrected). Sagittal, axial, and coronal views are shown. Each dot represents a region of the brain in which functional connectivity of that particular region to its connecting region was reduced in VPT children. Colour of dots corresponds to Fig. 1 (θ = red, α = yellow, β = green, low γ = blue, high γ = purple).

Whole-brain, global functional connectivity was reduced in very preterm compared to full-term born children (*p* < 0.01, corrected, Fig. 1). This reduction was observed in all five analysed frequency bands. To delineate the extent of disrupted network organization in the very preterm versus full-term groups, we applied a non-parametric statistical approach, NBS. Across five frequency bands, we identified 12 networks with reduced connectivity in the very preterm group, hereinafter referred to as ‘Network 1–12’ (*p* < 0.0005 corrected, Fig. 2, Table 4). We did not identify any networks with significantly increased connectivity in the very preterm-born group.

Some networks expressed densely anatomically focused reductions in connectivity, whereas other networks exhibited a more distributed pattern. Networks such as those shown for theta (i.e. Networks 1 and 2) and alpha (i.e. Network 5) were densely confined, involving multiple focal connections between adjacent lobes. In Network 1, the reduced connectivity was anchored in the frontal region, whereas in Network 5, diminished connectivity was prominent in occipital regions and extended to the basal ganglia and the dorsal visual stream. Very preterm children also demonstrated decreased connectivity encompassing more distributed networks such as those in the alpha (i.e. Network 4) and beta (i.e. Network 7), and high-gamma frequencies. These diffuse network differences encompassed connections involving the frontal lobes and midbrain regions.

### 3.3 Brain–behaviour relations

In the PLS analysis, we found no significant correlations between the global functional connectivity in any of the five frequencies and the neuropsychological scores in the very preterm-born children (*p* > 0.05, corrected).

## 4 Discussion

Employing a novel, whole-brain analysis of neurophysiological network connectivity, we present the first source-resolved evidence for reduced resting state network synchrony expressed across multiple temporal scales in very preterm-born children. This study confirms that, at school age, resting neural synchrony is disrupted at multiple frequencies in children born very preterm. We also delineate 12 spatially constrained networks that contribute to these global reductions in functional connectivity. The majority of the 12 networks encompass brain regions corresponding to established structural and resting state networks implicated in executive functioning. In addition, several networks segregate into specific frequency bands involved in attention and working memory. Such findings suggest the possibility that disconnection of these networks may contribute to developmental difficulties associated with very preterm birth.

### 4.1 Subplate and cortico-basal ganglia-thalamo-cortical loop

Atypical development of white matter in very preterm-born children has been shown to involve reduced thalamocortical connectivity in preterm infants (Ball, 2013) which is associated with worse cognitive outcome in childhood (Ball, 2015). Thalamocortical interactions are known to play a critical role in the generation of neural oscillations supporting neurophysiological network interactions and cognition (Hughes and Crunelli, 2005; Ribary, 2005). Adverse neonatal experience may impact the development of thalamocortical systems via influences on the subplate, which is key in establishing thalamocortical circuits. This may cause long-lasting impact on neurophysiological oscillations and networks subserving brain function and cognitive abilities. In support of this view, slowing of spontaneous MEG oscillations, which have been associated with disturbance of thalamocortical interactions (Llinas et al., 1999; Schulman, 2011), has been reported in school age children born very preterm (Doesburg, 2011b) and is associated with worse cognitive outcome in this group (Doesburg et al., 2013).

Thalamic input and thalamocortical interactions with the limbic system are thought to be modulated by theta band oscillations, and to be important in successful memory processing (Buzsáki, 2005; Siegle and Wilson, 2014). We identified one network (Network 3) in the theta band which encompassed the thalamus and other limbic structures, which suggests that very preterm-born children may demonstrate disrupted information transfer that may disrupt memory encoding and working memory processes.

In the gamma frequency range, we found two Networks (9 and 11) which included connections within the thalamocortical system as well as connections to the basal ganglia. The relation between cortex, the basal ganglia, and the thalamus is thought to be anatomically and functionally organized as the cortico-basal ganglia-thalamo-cortical loop (Cummings, 1993) allowing simultaneous processing of cognitive, sensorimotor and motivational information (Alexander and Crutcher, 1990). Structural connectivity within this loop has been reported to be reduced in school age children born extremely preterm with intrauterine growth restriction (Fischi-Gómez, 2015), and is thought to reflect a biological blueprint of less efficient simultaneous information processing seen in these populations. Gamma band synchrony has been associated with perceptual binding at early levels of sensory processing, attention, and working memory (Tallon-Baudry and Bertrand, 1999; Fries et al., 2001), and is purported to be involved in top-down modulation of sensory signals and large-scale integration of distributed neural networks (Uhlhaas, 2009). If functional coupling among regions of the cortex, thalamus and basal ganglia is interpreted to represent feed-forward and/or feedback activity, these results suggest that information transfer along these pathways follows ill-formed connections in very preterm children, possibly due to reduced structural brain connectivity. Future investigations using structural and functional data obtained in the same preterm cohort will help clarify such structure–function relations.

### 4.2 Executive and control networks

Cognitive control is a complex, multi-system process that appears to involve two distinct networks: a frontoparietal network (FPN), which serves as an adaptive control network, as well as a cingulo-opercular network (CON), which shows sustained activity across task (i.e. see Dosenbach, 2007). These two functional networks are hypothesized to support top-down control of executive functioning, and atypical functional connectivity within these networks may underlie cognitive deficits (for example, in schizophrenia (Meyer-Lindenberg, 2010; Repovs et al., 2011)). We found decreased neural synchrony in very preterm children in four networks (Network 4 in alpha, Network 7 in beta, and Networks 9 and 11 in gamma), which overlap with the FPN and CON. These disconnected networks include core hub regions involved in cognitive control, namely, the dorsolateral prefrontal cortex (listed as SFG in Table 4) in both Networks 4 and 9, the insula in Network 4, and the anterior cingulate (listed as ACG in Table 4) in Networks 7 and 11. The dorsolateral prefrontal cortex is thought to maintain neural representations of task-related goals (Miller and Cohen, 2001), while the anterior insula communicates with multiple large-scale networks to facilitate the processing of information marked as salient for attention and working memory processes (Menon and Uddin, 2010). The anterior cingulate has been shown to facilitate outcome-monitoring and conflict resolution during task (Botvinick et al., 2004).

In a task-based EEG-fMRI study requiring sustained vigilance as a marker of sustained alertness, alpha oscillations in EEG were found to correspond to neural activity in the CON network found in fMRI (Sadaghiani, 2010). Alpha band oscillations are understood to play a critical role in inhibition (Klimesch et al., 2007) and therefore a decrease in alpha synchrony in the CON (Network 4) in the very preterm children may reflect decreased ability to suppress distraction, or reduced task ability in processes requiring selective attention or inhibition. Whether these networks are truly similar in nature to those identified in fMRI studies is questionable given that the networks encompass association regions outside the defined set of frontoparietal and cingulo-opercular networks. A possible explanation is that the maturation of these executive control systems in very preterm-born children at school age is disrupted, and therefore this network demonstrates less segregation and integration than what is seen in term-born children.

### 4.3 Working memory and the prefrontal cortex in theta frequency, and visuospatial abilities in alpha frequency

Sustained attention relies on frontomedial theta oscillations, whereas selective excitation and inhibition of cognitive processing occurs through gamma and alpha oscillations, respectively (Womelsdorf and Fries, 2007). In electrophysiological studies involving working memory, these theta oscillations localize to the dorsomedial prefrontal and anterior cingulate cortices (Tsujimoto et al., 2006; Roberts et al., 2013; Hsieh et al., 2011). Activity among cortical areas involved in working memory was less synchronized in the very preterm group, notably in frontal, parietal and temporal lobes. Specifically, very preterm children showed reduced connectivity among prefrontal areas (such as the dorsolateral prefrontal cortex) and the inferior parietal lobule and middle temporal gyrus in theta (Networks 1 and 2), alpha (Network 4), beta (Network 6) and low-gamma (Network 9).

Altered cortical activation of prefrontal cortex during language processing has also been described in preterm-born adolescents (Frye, 2010), and atypical activation and reduced functional connectivity involving prefrontal cortex, involving theta oscillations, have been reported in school age children born very preterm (Moiseev et al., 2015). Network 1 supports and extends such findings from task-based studies. These prior results indicate that information transfer in distributed neural systems supporting working memory may be diminished in school age children born very preterm, and our present findings suggest this may also be reflected in intrinsic brain activity.

Alpha rhythms support vision and perception (Palva and Palva, 2007). Throughout development, very preterm children have been shown to be at high risk for cognitive impairment and educational underachievement, especially in domains related to working memory/executive functions and visuospatial abilities (Aarnoudse-Moens et al., 2009b; Anderson et al., 2004). These cognitive skills involve dorsal visual circuits, and are abnormal in task-based investigations in the very preterm population (Ment et al., 2009). Using MEG, reduced network synchronization at the sensor level during task performance has been linked to visual–perceptual abilities in very preterm children (Doesburg, 2011). Our results in the alpha frequency (Network 5) support and extend these findings.

### 4.4 Advantages and limitations

An important advantage of our study is that investigating neural oscillations offers comparable indexes of rhythmic activity across different species and spatial scales. This allows for direct comparison between human data assessed with MEG and invasive recordings in animals as well as fMRI. This can also facilitate understanding mechanistic relations between physiological disruptions due to preterm birth and behavioural and cognitive phenomena.

The analysis approach we used has also been used to investigate atypical network connectivity in adolescents with autism spectrum disorder (Ye et al., 2014). Our study further demonstrates the robustness of using resting-state MEG recordings as a new approach to examine alterations of the neurophysiological connectome in clinical child populations. Elucidation of relations between spontaneous network synchrony, neonatal variables and cognitive outcome will require further study with a larger cohort, ideally longitudinally followed from birth.

Numerous factors including targeted early intervention may impact neurodevelopment in very preterm-born children. Due to the cross-sectional nature of our study, we did not have access to interventional history of the children. To maximize the current utility in the clinical context, future studies of resting state MEG data need to be done with knowledge of complementary clinical and social information.

It is feasible that the reduced neurophysiological network interactions identified in the present study contribute to wide-ranging functional deficits. Although frequency-specific topology of connectivity reductions is intriguing, links between such brain alterations and neurocognitive outcomes remain speculative in terms of the results of the present study. A continuing central challenge is to determine how the longitudinal trajectories of specific functional systems relate to cognition and behaviour in these very preterm-born school age children.

## 5 Conclusions

We provide the first evidence that very preterm-born children at school age express large-scale reductions in neurophysiological resting brain connectivity, and that these effects occur in brain networks underlying cognitive functions frequently reported deficit in this population. Our findings demonstrate the potential of MEG for investigating brain network integration and segregation across multiple frequency domains pertinent to cognitive function and its relation to cognitive long-term outcome. The presence of the focally disconnected networks identified in this study in a task-free state at these frequencies, and concentrated in specific anatomical brain networks, offers novel clues to understanding the neurophysiological underpinnings that place very preterm-born children at risk for cognitive difficulties.

## Figures and Tables

**Table 1:** Gestation, age, and sex in very preterm and full term control children included in the study.

	Very preterm	Full term
*N*	17	17
Boys, *n*	9	9
Age at assessment, years (SD)	10.2 (2.0)	10.2 (1.9)
Birth weight g (SD)	1077.5 (286)	–
Gestation, weeks (SD)	28.0 (2.0)	–
32 weeks, *n* (%)	1 (6)	–
31 weeks, *n* (%)	1 (6)	–
30 weeks, *n* (%)	2 (12)	–
29 weeks, *n* (%)	2 (12)	–
28 weeks, *n* (%)	5 (29)	–
27 weeks, *n* (%)	2 (12)	–
26 weeks, *n* (%)	4 (23)	–

**Table 2:** Scores for term-born controls and very preterm children on standardized and neuropsychological assessment.

Dependent variable	Very preterm, mean (SD)	Full term, mean (SD)	*p* value (FDR corrected)	Effect size (Cohen's d)
*WASI*				
*n*	17	16		
Two-subtest IQ	106.7 (12.6)	115.9 (12.2)	.178	−0.77

*NEPSY II*				
*n*	17	17		
Animal sorting, scaled score	8.9 (2.4)	10.7 (4.1)	.218	−0.52
Inhibition — naming combined scaled score	8.8 (3.0)	10.0 (3.8)	.391	−0.33
Inhibition — inhibition combined scaled score	9.4 (3.6)	11.1 (3.2)	.226	−0.49
Inhibition — switching combined scaled score	9.6 (2.5)	11.5 (4.0)	.201	−0.57
Affect recognition Total scaled score	11.4 (1.3)	11.5 (2.1)	.838	−0.10
*n*	17	12		
Theory of mind Total score	22.5 (2.6)	24.2 (3.1)	.908	−0.04

*WMTB-C*				
*n*	17	16		
Forward digit recall	102.4 (13.2)	110.5 (20.5)	.247	−0.46
Backward digit recall	90.3 (10.3)	101.6 (20.5)	.178	−0.69

*BRIEF*				
*n*	16	15		
GEC	49.3 (9.7)	42.5 (8.0)	.178	0.74
BRI	47.5 (10.3)	42.1 (6.2)	.201	0.61
MCI	49.9 (9.1)	43.5 (9.2)	.178	0.69

**Table 3:** The 90 brain regions (left and right for each region) corresponding to the AAL atlas, their MNI coordinates, and short-form abbreviations.

Region name	Abbreviation	MNI coordinates					
Left			Right		
X	Y	Z	X	Y	Z
Precentral gyrus	PreCG	−38.65	−5.68	50.94	41.37	−8.21	52.09
Superior frontal gyrus, dorsolateral	SFG.L	−18.45	34.81	42.2	21.9	31.12	43.82
Superior frontal gyrus, orbital part	SFGorb	−16.56	47.32	−13.31	18.49	48.1	−14.02
Middle frontal gyrus	MFG	−33.43	32.73	35.46	37.59	33.06	34.04
Middle frontal gyrus, orbital part	MFGorb	−30.65	50.43	−9.62	33.18	52.59	−10.73
Inferior frontal gyrus, opercular part	IFGoper	−48.43	12.73	19.02	50.2	14.98	21.41
Inferior frontal gyrus, triangular part	IFGtri	−45.58	29.91	13.99	50.33	30.16	14.17
Inferior frontal gyrus, orbital part	IFGorb	−35.98	30.71	−12.11	41.22	32.23	−11.91
Rolandic operculum	ROL	−47.16	−8.48	13.95	52.65	−6.25	14.63
Supplementary motor area	SM A	−5.32	4.85	61.38	8.62	0.17	61.85
Olfactory cortex	OLF	−8.06	15.05	−11.46	10.43	15.91	−11.26
Superior frontal gyrus, medial	SFGmed	−4.8	49.17	30.89	9.1	50.84	30.22
Superior frontal gyrus, medial orbital	SFGmorb	−5.17	54.06	−7.4	8.16	51.67	−7.13
Gyrus rectus	REC	−5.08	37.07	−18.14	8.35	35.64	−18.04
Insula	INS	−35.13	6.65	3.44	39.02	6.25	2.08
Anterior cingulate and paracingulate gyri	ACG	−4.04	35.4	13.95	8.46	37.01	15.84
Median cingulate and paracingulate gyri	DCG	−5.48	−14.92	41.57	8.02	−8.83	39.79
Posterior cingulate gyrus	PCG	−4.85	−42.92	24.67	7.44	−41.81	21.87
Hippocampus	HIPP	−25.03	−20.74	−10.13	29.23	−19.78	−10.33
Parahippocampal gyrus	PHG	−21.17	−15.95	−20.7	25.38	−15.15	−20.47
Amygdala	AMYG	−23.27	−0.67	−17.14	27.32	0.64	−17.5
Calcarine fissure and surrounding cortex	CAL	−7.14	−78.67	6.44	15.99	−73.15	9.4
Cuneus	CUN	−5.93	−80.13	27.22	13.51	−79.36	28.23
Lingual gyrus	LING	−14.62	−67.56	−4.63	16.29	−66.93	−3.87
Superior occipital gyrus	SOG	−16.54	−84.26	28.17	24.29	−80.85	30.59
Middle occipital gyrus	MOG	−32.39	−80.73	16.11	37.39	−79.7	19.42
Inferior occipital gyrus	IOG	−36.36	−78.29	−7.84	38.16	−81.99	−7.61
Fusiform gyrus	FUSI	−31.16	−40.3	−20.23	33.97	−39.1	−20.18
Postcentral gyrus	PoCG	−42.46	−22.63	48.92	41.43	−25.49	52.55
Superior parietal gyrus	SPG	−23.45	−59.56	58.96	26.11	−59.18	62.06
Inferior parietal lobule	IPL	−42.8	−45.82	46.74	46.46	−46.29	49.54
Supramarginal gyrus	SMG	−55.79	−33.64	30.45	57.61	−31.5	34.48
Angular gyrus	ANG	−44.14	−60.82	35.59	45.51	−59.98	38.63
Precuneus	PCUN	−7.24	−56.07	48.01	9.98	−56.05	43.77
Paracentral lobule	PCL	−7.63	−25.36	70.07	7.48	−31.59	68.09
Caudate nucleus	CAU	−11.46	11	9.24	14.84	12.07	9.42
Lenticular nucleus, putamen	PUT	−23.91	3.86	2.4	27.78	4.91	2.46
Lenticular nucleus, pallidum	PAL	−17.75	−0.03	0.21	21.2	0.18	0.23
Thalamus	THA	−10.85	−17.56	7.98	13	−17.55	8.09
Heschl gyrus	HES	−41.99	−18.88	9.98	45.86	−17.15	10.41
Superior temporal gyrus	STG	−53.16	−20.68	7.13	58.15	−21.78	6.8
Temporal pole: superior temporal gyrus	TPOsup	−39.88	15.14	−20.18	48.25	14.75	−16.68
Middle temporal gyrus	MTG	−55.52	−33.8	−2.2	57.47	−37.23	−1.47
Temporal pole: middle temporal gyrus	TPOmid	−36.32	14.59	−34.08	44.22	14.55	−32.23
Inferior temporal gyrus	ITG	−49.77	−28.05	−23.17	53.69	−31.07	−22.32

**Table 4:** Disconnected networks at each frequency band and their participating brain regions in very preterm children in comparison to full term controls. Refer to Table 3 for full region names.

Network (very preterm < full term, *p* < 0.0005)	Frequency band	Region names
1	Theta (θ)	L.PreCG, R.PreCG, L.SFG, R.SFGorb, R.MFGorb, R.SMA, LSFGmed, R.SFGmorb, R.REC, L.ACG, R.PoCG, R.ANG, R.PCL, R.TOPsup
2	R.SFG, R.MFG, R.IFGorb, L.OLF, R.OLF, L.DCG, R.PCG, R.HIPP, R.PHG, L.PCL, R.PUT, R.HES, L.STG, L.TOPsup, R.MTG, R.TOPmid
3	L.MFG, L.SMA, L.SFGmorb, R.INS, L.AMYG, R.CUN, L.THA, L.TOPmid
4	Alpha (α)	R.SFG, L.INS, R.PoCG, R.IPL, L.TOPsup, L.MTG
5	L.SMA, L.HIPP, L.CUN, L.LING, R.LING, R.SOG, L.MOG, L.IOG, L.SPG, L.IPL, L.ANG, L.PCUN, R.PCUN, L.CAU, L.PUT, R.STG
6	Beta (β)	L.MFG, R.MFG, L.IFGorb, R.ROL, R.SFGmed, L.SFGmorb, R.AMYG, R.PAL, L.HES, R.HES, R.STG
7	L.MFGorb, L.ACG, L.PHG, R.PoCG
8	L.SMA, R.SMA, L.REC, R.PHG, L.SOG, R.SMG
9	Low gamma (λ)	L.SFGorb, R.SFGorb, L.ROL, R.ROL, L.SFGmed, L.SFGmorb, R.SOG, L.IPL, L.PUT, R.THA, R.HES
10	High gamma (λ)	R.PreCG, L.ROL, R.ACG, R.PCL
11	L.IFGorb, R.ROL, L.REC, L.INS, L.ACG, R.DCG, L.PCG, L.AMYG, R.CAL, R.LING, R.SOG, R.FUSI, R.PoCG, L.IPL, R.IPL, L.SMG, R.SMG, R.CAU, R.PUT, R.THA, L.HES, L.STG, L.TOPsup, L.TOPmid, R.TOPmid
12	L.OLF, R.INS, L.CAL
