# Supplementary material for: Disconnected neuromagnetic networks in children born very preterm: Disconnected MEG networks in preterm children
Source: Neuroimage Clin. 2015 Sep 1;9:376–84. doi: 10.1016/j.nicl.2015.08.016 (PMC4589841; doi:10.1016/j.nicl.2015.08.016)

**Supplementary Materials**

*Ye et al., Disconnected neuromagnetic networks in children born very preterm*

Supplementary Methods

*1.0 Global power computation*

Following beamformer projection, oscillatory amplitude envelopes of the reconstructed time series were estimated via computation of the absolute value of the analytic signal, which was found using a Hilbert transform. This yielded an estimate of the instantaneous signal amplitude for each source, termed the Hilbert envelope. We then averaged over all sources to obtain one value representing the global power for each individual, at each frequency band. Group differences at each frequency were evaluated using Mann-Whitney U tests. Bonferroni correction was applied to account for multiple comparisons across the frequency bins studied (i.e. threshold for significant, p<(0.05/N), where N = 5).

*2.0 Neuropsychological Testing*

Each participant received a neuropsychological battery consisting of subtests to evaluate general intelligence, working memory, executive functioning, and social perception. Altogether, participants received 12 scores derived from several neuropsychological assessments: Wechsler Abbreviated Scale of Intelligence (WASI, 1 score), Working Memory Test Battery for Children (WMTB-C, 2 scores) [Pickering & Gathercole, 2001], NEPSY-II (6 scores) [Korkman M, Kirk U, Kemp SL. NEPSY II, 2nd ed. San Antonio: PsychCorp/Pearson Clinical Assessment, 2007.], and Behavior Rating Inventory of Executive Functioning (BRIEF, 3 scores). Each test is described in more detail below. All scores reported in Table 2 were standardized to age norms.

2.1 WASI

We assessed general intellectual ability as assessed by the WASI. We administered the two subtest version of the WASI (Vocabulary and Matrix Reasoning) which provides a brief and reliable estimate of the child’s intellectual functioning. In Table 2, we report age-standardized scores with a mean of 100 and standard deviation of 15.

2.2 WMTB-C

We administered two subtests from the WMTB-C, which consisted of the Digit Recall test to evaluate phonological short-term memory and the Backwards Digit recall test to evaluate complex memory span. The WMTB-C is designed to measure working memory abilities of children aged 5 to 15 years. Both the Digit Recall and the Backwards Digit Recall test have the same structure. In the Digit Recall test, the child is required to recall a verbally spoken sequence of digits in the order they were presented. In the backwards digit recall test, the child is required to recall a sequence of spoken digits in reverse order. We report age-normed scores which have a mean of 100 and standard deviation of 15 in Table 2.

2.3 NEPSY-II

The NEPSY-II is a standardized neuropsychological battery for children aged 3-16 years that assesses functioning across six domains: executive functioning and attention, memory and learning, sensorimotor functioning, language, visuospatial processing, and social perception. For the purpose of this study, we administered four subtests from the NEPSY-II battery used to test the domains of Executive Functioning and Attention (animal sorting and inhibition) and Social Perception (affect recognition and theory of mind). The children received a teaching example to ensure that they understood the task. All subtests yield a mean standard score of 10 with a standard deviation of 3 (see Table 2).

2.3.1 Animal Sorting subtest

This subtest is under the domain of Executive Functioning and Attention. Animal sorting assesses initiation, cognitive flexibility, and self-monitoring. The child sorts cards into two groups of four cards each using various self-initiated sorting criteria. An error occurs when the child completes a card sort that is incorrect, or the child repeats a card sort. The score integrates aspects of correct sorting with cognitive control of the sorting behavior by combining the Total Correct Sorts and Total Errors scores, and weights accuracy of performance over the penalization of errors.

2.3.2 Inhibition subtest

This subtest is under the domain of Executive Functioning and Attention and assesses the ability to inhibit automatic responses in favour of novel responses and the ability to switch between response types. In this task, children are first shown a series of black and white circles and squares which they are asked to name as fast as possible. Next, children are shown the identical images and are required to provide the opposite name for each. In the Switching section of the subtest, the child is shown black and white arrows or shapes (circles and squares). The child is then asked to state the opposite direction if the arrow is white, and the correct direction if the arrow is black. The conditions are repeated with shapes. An error occurs when a child provides an incorrect response or self corrects his/her response. Each score integrates the total number of errors made (uncorrected and self-corrected) as well as total completion time for that condition. We report three age-adjusted scaled scores in Table 2: Inhibition-Naming Combined Scaled Score, Inhibition-Inhibition Combined Scaled Score, and Inhibition-Switching Combined Scaled Score.

2.3.3 Affect recognition subtest

This subtest from the NEPSY-II is under the domain of Social Perception. This subtest assesses the ability to discriminate among common facial expressions (happy, sad, anger, fear, disgust, and neutral affect) in four different tasks. The tasks require the child to compare expressions on children’s faces. In one task, the child decides whether or not two photographs depict faces with the same expression. In a second task, they determine which two faces have similar expressions from a selection of 3-4 cards. In a third task, the child selects one of four faces that depicts the same affect as a face at the top of the page. In the final task, the child is briefly shown a face and from memory, selects two photographs that depict the same affect as the face previously shown. We report the Affect Recognition Total Scaled Score in Table 2, which provides a general measure of the child’s ability to recognize facial expressions.

2.3.4 Theory of Mind subtest

This is a subtest under the domain of Social Perception. This subtest is designed to assess the ability to understand mental functions such as belief, intention, deception, emotion, imagination, and pretending. It also assesses the ability to understand that others have their own thoughts, ideas, and feelings that may be different from one’s own and the ability. In the Verbal task, the child is read various scenarios or shown pictures and is asked questions that require knowledge of another individual’s point of view to answer correctly. In the Contextual task, the child is shown a picture depicting a social context and asked to select a photograph from four options that depicts the appropriate affect of one of the people in the picture. We report the Theory of Mind Total Score in Table 2. A low Total Score indicates difficulties in understanding other’s perspectives.

2.4 BRIEF

We administered the Parent form of the BRIEF to assess everyday executive abilities. This form consists of 86 items that evaluate the areas of emotion regulation, behaviour regulation, and metacognition. The responses give rise to scores on eight non-overlapping clinical scores on which T scores are obtained. Then, two summary indices (the Behavioural Regulation Index, or BRI, and the Metacognition Index, or MI) are calculated from the clinical scales. Finally, the scores on the BRI and MI are combined to yield a Global Executive Composite (GEC). T scores are normative scores with a mean of 50 and a standard deviation of 10. T scores of 65 or higher on a particular scale or index indicate potential clinical significance. We report three scores (the BRI, MI, and GEC), which is considered a meaningful summary measure of overall executive functioning.

Supplementary Results

*Global power analysis*

There were no significant differences (*p* > 0.05, Bonferroni corrected) in global power across the entire brain between very preterm born children and full term born children in all frequency bins (**Supplementary Figure 1**).

**Supplementary Figure 1**. Average power (amplitude) across the whole brain for each group and frequency range. Values represent the average Hilbert envelope of 90 regions across the cortex and sub-cortex. VPT = very preterm born children, FT = full term born children.


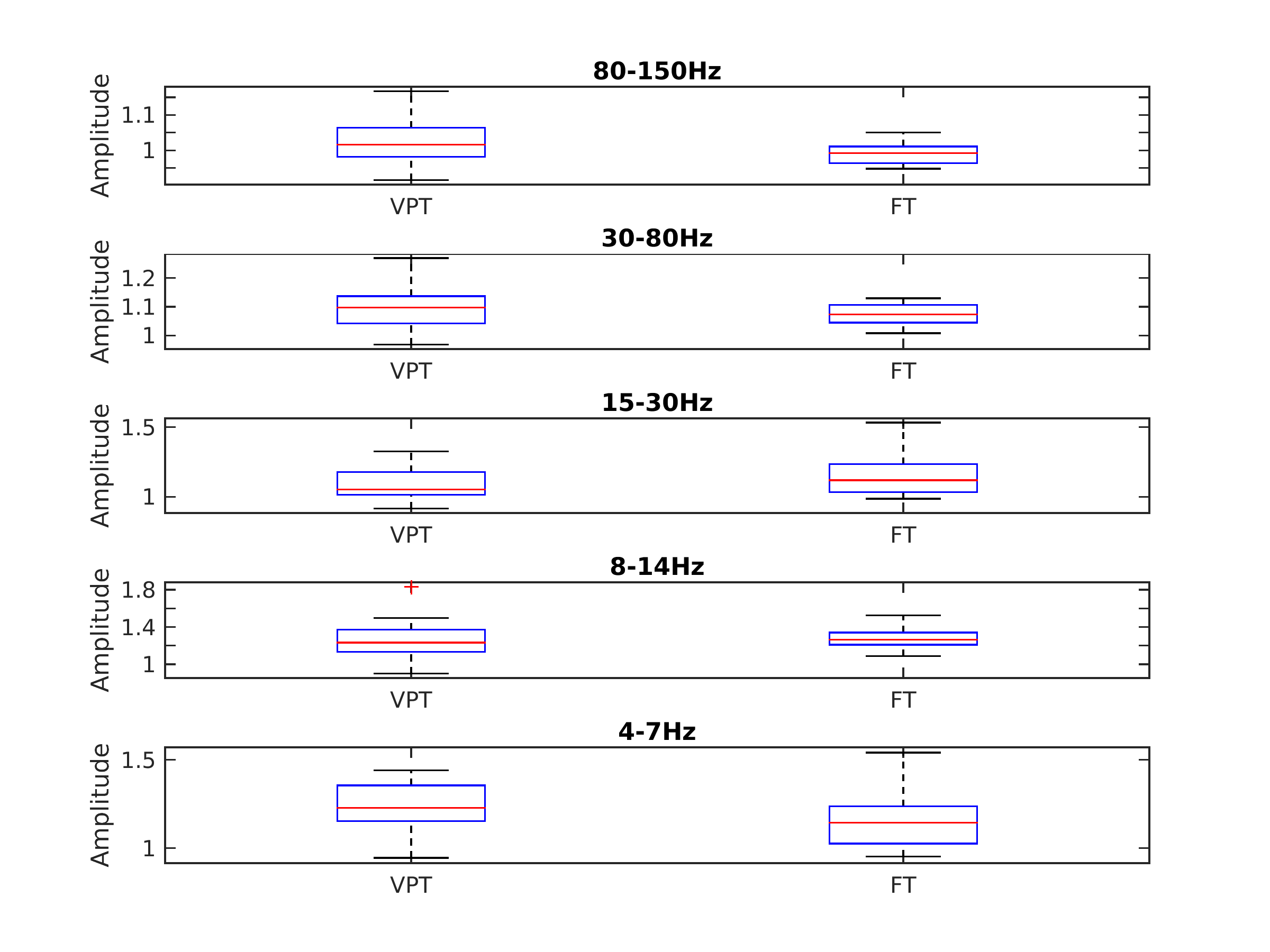

Supplement: Supplementary file 1 — Supplementary material. [file mmc1.docx]
